# Is Quarantine for COVID-19 Pandemic Associated with Psychological Burden in Primary Ciliary Dyskinesia?

**DOI:** 10.3390/ijerph17218099

**Published:** 2020-11-03

**Authors:** Maria Pia Riccio, Melissa Borrelli, Maria Teresa Fioretti, Margherita Del Bene, Carmela Bravaccio, Marco Poeta, Francesca Santamaria

**Affiliations:** Department of Translational Medical Sciences, Federico II University, 80131 Naples, Italy; piariccio@gmail.com (M.P.R.); melissa.borrelli@unina.it (M.B.); m.teresafioretti@hotmail.it (M.T.F.); margherita.delbene@gmail.com (M.D.B.); carmela.bravaccio@unina.it (C.B.); po3ta.89@gmail.com (M.P.)

**Keywords:** primary ciliary dyskinesia, severe acute respiratory syndrome due to coronavirus 2, COVID-19 pandemic, psychological burden, parental stress

## Abstract

Background: Information on psychological impact of COVID-19 quarantine in primary ciliary dyskinesia (PCD), a chronic disorder with recurrent pulmonary exacerbations, is lacking. Psychological well-being was prospectively assessed during COVID-19 lockdown in Italy in a PCD population. Methods: we recruited 27 PCD patients and 27 healthy controls. To assess psychological well-being, psychological general well-being index and parenting stress index-short questionnaires were administered to participants ≥15 years-old and to mothers of participants <15 years-old, respectively. The PCD exacerbations since outbreak onset and frequency of quarantine weekly chest physiotherapy were compared to the same period of 2019. Outcomes: 70% of PCD mothers and 90% of PCD patients did not show parental stress levels or distress levels, respectively, and these groups showed no significant difference in stress compared to controls. The PCD pulmonary exacerbations occurred less frequently and weekly chest physiotherapy sessions significantly increased compared to the same period during 2019 (*p* < 0.05). Interpretation: During COVID-19 quarantine, a PCD population showed psychological well-being. Low exacerbation rate, explained by lower infectious exposure or improved compliance to chest physiotherapy, likely contributed to psychological well-being. Evaluating psychological burden and parental stress is a valuable tool for measuring the emotional impact of PCD and improving PCD medical care.

## 1. Background

Originating in December 2019 as a cluster of unexplained pneumonia in China, the novel severe acute respiratory syndrome due to coronavirus 2 (SARS-CoV-2), designated as coronavirus disease 2019 (COVID-19), rapidly reached the level of a global pandemic [1]. The clinical spectrum of adult COVID-19 ranges from pauci- or asymptomatic forms to acute respiratory distress syndrome, and in several cases, multi-organ failure and death have been described [2]. Incidence of pediatric COVID-19 is lower than adult disease, with fewer critical cases, and very few deaths [3].

Pre-existing chronic conditions might pose a threat to individuals with severe COVID-19 symptoms [4]. However, the COVID-19 course was not significantly different for patients with non-allergic asthma and cystic fibrosis (CF) from the general population [5,6].

Primary ciliary dyskinesia (PCD; MIM244400), a rare genetic disorder, is characterized by impaired mucociliary clearance and recurrent-to-chronic respiratory infections [7]. Development of bronchiectasis with progressive loss of pulmonary function, fertility issues, and situs viscerum inversus in 50% of cases (Kartagener syndrome) are the hallmarks of the condition [8]. Yet, in adolescence or young adulthood, PCD lung disease may progressively deteriorate and therefore PCD is considered a chronic entity [7]. No reports on COVID-19 in PCD exist at the current time.

Because of the massive outbreak of COVID-19 reported in Italy on last February [9], the Government adopted a plan of preventive measures to contain the viral spread [10]. According to the decree of the President of the Council of Ministers, the whole population was placed under lockdown and strict social isolation was imposed from 8 March 2020 up to 4 May 2020 [11].

Isolation has a considerable psychological impact on either infected individuals or those who keep distance for preventing infection [12]. An impaired quality of life has been also reported in literature during COVID-19 pandemic due to the social distancing [13,14]. Moreover, related lockdown measures could impact on mental health, due to isolation [15,16]. In addition to concerns associated with the diffusion of the outbreak, limited access to hospital facilities during the quarantine period could make patients with chronic disorders susceptible to psychological stress. As respiratory exacerbations result in PCD increased morbidity [8], concerns on COVID-19 are expected to impact patients’ psychological well-being, but information is lacking on this argument. The primary aim of our study was to prospectively assess the PCD patients’ psychological burden and parental stress levels compared to healthy people during the lockdown period in Italy.

## 2. Methods

A prospective study of a PCD population including either patients with PCD or their mothers was performed. Participants were compared to sex- and age-matched healthy subjects. Signed informed consent was obtained from all participants. The study was performed in accordance with the Declaration of Helsinki for Human Research and approved by the Ethical Committee, Federico II University, Naples (protocol no. 275/20).

## 3. Patients

At the Department of Translational Medical Science, University “Federico II”, Naples, Italy, a specialized center that provides care to children and adults with PCD was instituted in 1993. Patients are followed through scheduled ambulatory visits, which include sputum culture, spirometry, chest imaging (when needed), and hospital admission for intravenous antibiotic administration and/or treatment of medical/surgical complications. At our center, treatment of PCD consists of (a) airway clearance techniques at least twice each day aimed to facilitate clearance of mucus from the airways, i.e., nebulised hypertonic saline followed by chest physiotherapy (including percussion and vibration and/or postural drainage and/or autogenic drainage and/or active cycle breathing, with or without the aid of positive expiratory pressure devices and mechanical coughassist); and (b) antibiotic treatment in case of airway infectious exacerbation.

As soon as the notice of the President of the Council of Ministers that imposed the quarantine in Italy was received (8 March 2020) [11], we modified our policy of care to PCD patients. According to our hospital board, regular hospital visits, scheduled on a three-month basis, were switched to patients’ home-based management through remote assistance service in telemedicine. Telemedicine consisted of weekly telephone or email contacts between patients and PCD physicians. Treatment was confirmed and/or modified on the basis of individual needs. Patients could not access the hospital unless urgent consultations were required.

During the quarantine (8 March 2020–4 May 2020), after a telephone contact, 27 PCD patients were remotely enrolled. Inclusion criteria included PCD diagnosis according to the European Respiratory Society guidelines [17], active follow-up for at least 12 months and adherence to the study protocol after informed consent signature. Exclusion criteria included confirmed or suspected COVID-19 infection, PCD diagnosis made in 2020, concomitant chronic diseases or psychiatric/neurodevelopmental disorders, incomplete information availability, and the inability to give informed consent.

## 4. Study Design

The enrolled PCD patients or their parents received by email a questionnaire aimed to assess their psychological stress level in the quarantine period. We divided the PCD population in two age-based groups, <15 (Group A) and ≥15 years-old (Group B), respectively. The age limit was set at 15 years as we used a questionnaire on the self-perceived evaluation of psychological well-being, previously validated in subjects aged ≥15 years [18].

In Group A, PCD parents’ stress was assessed through the Italian version of the parenting stress index-short form questionnaire (PSI-SF) [19]. The PSI-SF is a standardized self-report tool that explores the stress levels of parents or caregivers of children [19,20]. PSI-SF is a 36-items with each item rated on a five-point Likert-type scale. The PSI-SF is organized into four subscales: parental distress (PD), parent-child dysfunctional interaction (P-CDI), difficult child (DC), defensive responding (DEF, which indicates likely response bias). It provides a total stress score index. High scores indicated high perceived stress in the parents. The PSI-SF provides both raw and percentile scores. Scores >85th percentile indicate ‘high stress level’; scores between 50th and 85th percentile indicate ‘symptomatic stress level’; finally, scores <50th percentile indicate ‘non-pathological stress levels’. The PSI-SF validity has been established in parents of children with PCD and other chronic medical conditions, such as diabetes or asthma [21,22]. In the current study, the PSI-SF was administered only to the mother, who usually spends more time with the children than the father.

In Group B, PCD patient psychological well-being was assessed through the Psychological General Well-Being Index (PGWBI) questionnaire, Italian version [23]. The PGWBI is a self-administered questionnaire that measures the level of subjective psychological well-being in participants ≥ 15 years [18]. It assesses the self-representations of intrapersonal affective or emotional states that reflects a sense of subjective well-being or distress. The PGWBI consists of 22 standardized items organized in 6 subscales: anxiety, depression, well-being, self-control, general health, and vitality. Responses for each item are evaluated on a six-point Likert-type scale (range 0–5). High scores indicate high well-being. The subscales sum provides a global index score of subjective well-being (range 0–110). A total score <60 suggests ‘severe distress’, whereas the ‘moderate distress’ and ‘no distress’ categories are defined by a global score of 60 to 72, and >72, respectively. The PGWBI provides both raw and percentile scores.

Subscales and global scores percentiles were defined according to gender and age as follows: scores <25th percentile indicate ‘severe distress’; scores between 25th and 50th percentile indicate ‘moderate distress’; scores between 50th and 75th percentile indicate ‘no distress’; finally, scores >75th percentile indicate ‘positive well-being’ [23]. The PGWBI and the PSI-SF have been previously validated in Italian [19,23].

When the study protocol was designed, it was decided to assess also: (a) the PCD airway infectious exacerbations rate occurring in PCD patients from the beginning of the outbreak in Italy. According to a recent publication, we resolved in advance to concentrate on infections occurring in the lower airways [24]. The PCD pulmonary infectious exacerbations occurring from February 1 to May 4, 2020 were compared to exacerbations registered in the medical records over the same period of the previous year (2019); (b) the number of chest physiotherapy per week performed by each patient during the quarantine period, which was compared to the number registered in the medical records in the same period of the previous year (2019). Data referring to 2020 were obtained by email or telephone interview to PCD patients or PCD mothers.

The PGWBI and the PSI-SF questionnaires were also administered to 27 age- and sex-matched controls, including 17 healthy subjects aged ≥ 15 years and the mothers of 10 healthy subjects aged less than 15 years, respectively.

Adherence to the measures imposed by the government was evaluated by administering the following questions to (a) adult workers (either PCD patients or controls): “During the lockdown period did you adopt the smart working model?”; (b) subjects with PCD or controls either attending school or university: “During the lockdown period did you/your kid stop attending school or university courses?”.

## 5. Statistical Analysis

Analyses were made with Graphpad Software, version 8.0.0 (San Diego, CA, USA). The Kolmogorov–Smirnov Test was used to evaluate normality distribution of data. Data were presented as mean (standard deviation (SD)) or median (interquartile range (IQR)), and categorical variables as frequency (%). The exact Fisher, T-student and Mann–Whitney tests were used to compare patients and controls and data of PCD patients between the two study periods (2019 and 2020). A two-sided *p* < 0.05 was considered significant.

## 6. Results

PCD patients included 10 subjects aged <15 years, and 17 cases aged ≥15 years. Demographic and clinical characteristics are summarized in Table 1. 10 healthy participants aged <15 years (70% males; mean age 10.3 years (SD 3)), and 17 healthy participants ≥15 years (65% males;, mean age 34.5 years (SD 12.6)) were recruited as control group.

Patients and families with PCD, as well as controls and their families, strictly observed the stringent preventive measures ordered by the government. All adults adopted the smart working model and all students attended online school or university courses at home. None of the patients had confirmed or suspected COVID-19 or had undergone medical consultations related to COVID-19.

All participants, including PCD patients, mothers and controls sent back the questionnaires on 5 May 2020.

**Figure 1:**
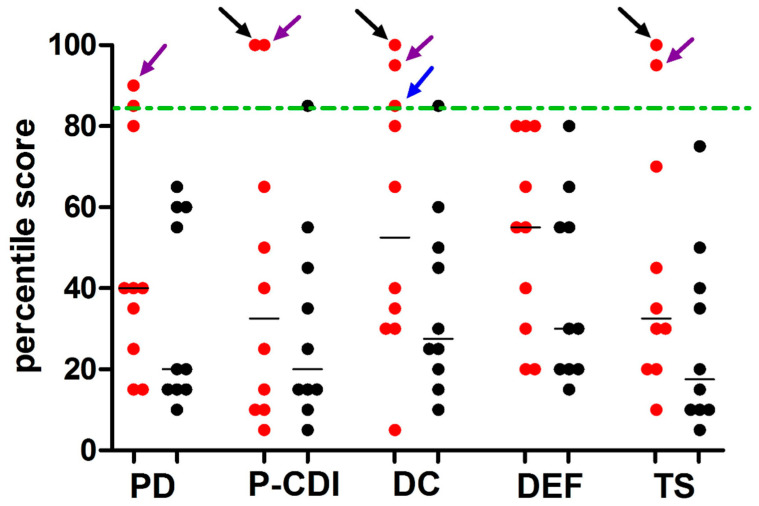
Distribution of PSI-SF percentile scores of subscales and total score from PCD mothers and controls. Legend: the parenting stress index-short form questionnaire (PSI-SF) was used to explore the stress levels in the mothers of primary ciliary dyskinesia (PCD) patients (n = 10) and of healthy controls (n = 10) younger than 15 years. The PSI-SF is organized intofour subscales: parental distress (PD), parent-child dysfunctional interaction (P-CDI), difficult child (DC), defensive responding (DEF). Each point (red and black for PCD patients and controls, respectively) represents the percentile score taken from a single participant. Horizontal bars represent the median values. Dashed lines indicate the 85th percentile (high stress level). Only 3 PCD mothers presented a pathologic percentile score: one (purple arrow) reported pathologic percentile score in PD, PCDI, DC subscales and in total score (TS); one (black arrow) reported pathologic percentile score in PCDI, DC and TS; one (blue arrow) reported a score equal to 85th percentile only in DC subscale. Both in subscales score and total score, the percentiles were not significantly different between patients and healthy controls (*p* > 0.05).

PSI-SF questionnaire was administered to PCD mothers and to mothers of healthy participants younger than 15 years. Results showed that in 7/10 cases (70%), the total scores were below the 50th percentile and, therefore, did not suggest a stress condition (Table 2). Only in 3/10 PCD mothers (30%) did the total scores fall above the 50th percentile, indicating parental stress. Of all mothers in control group, only 1/10 case (10%) showed a total score between the 50th and 85th percentile. In the remaining cases (90%) the total scores were below the 50th percentile. The percentage of PCD and control group mothers with different categories of stress level (high; symptomatic stress; non-pathological stress) were not significantly different (*p* > 0.05). The PSI-SF mean scores related to total stress and subscales (PD, P-CDI, DC, DEF) in PCD patients’ mothers (Group A) were not significantly different than the same scores in controls’ mothers (Table 2). However, 3/10 PCD mothers (30%), and only 1/10 mother in the control group (10%), reported a DC score equal to or higher than 85th percentile (Figure 1).

**Figure 2:**
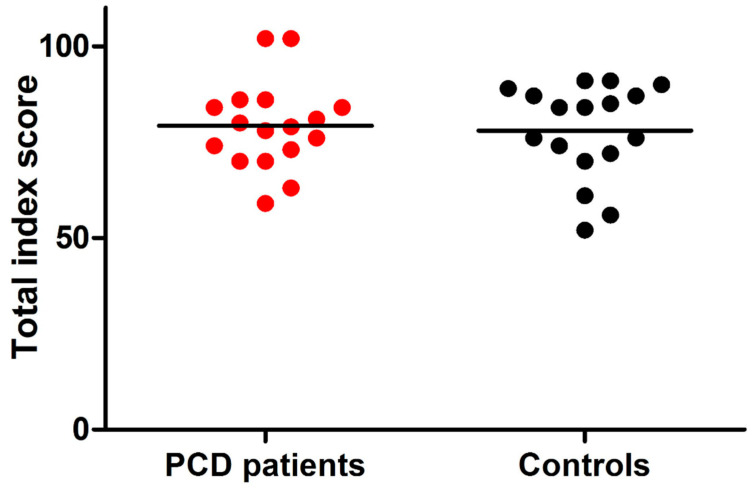
Distribution of PSGWBI total index score from PCD patients and controls. Legend: Psychological general well-being index (PSGWBI) questionnaire was administered in primary ciliary dyskinesia (PCD) patients (n = 17) and healthy controls (n = 17) ≥15 years. The PSGWBI is organized into subscales, the subscales sum provides a total index score for subjective well-being (range 0–110). Considering ‘distress’ as the reverse of well-being, a total score <60 suggests ‘severe distress’, while the ‘moderate distress’ and ‘no distress’ categories are defined with a total score between 60 and 72, and >72, respectively. Each point (red and black for PCD patients and controls, respectively) represents the total index score obtained by a single subject. Horizontal bars represent the mean values. The dashed line represents the total index score of 60. Only one PCD patient and two controls presented pathological scores. There were no significant differences of total index score between PCD patients and healthy controls (*p* > 0.05).

**Figure 3:**
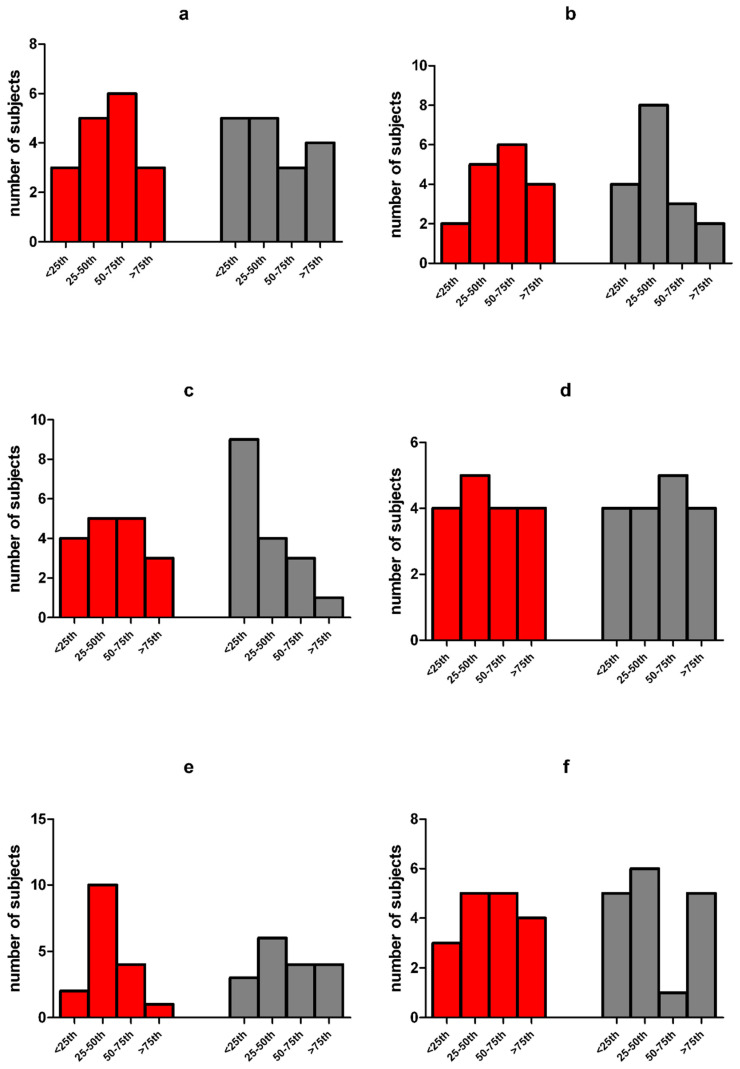
(**a**–**f**) Distribution of PCD patients and controls in the classes of percentile of PSGWBI subscales. Legend: the psychological general well-being index (PSGWBI) questionnaire was administered to primary ciliary dyskinesia (PCD) patients (n = 17) and healthy controls (n = 17) older than 15 years. The PSGWBI is organized in six subscales: (**a**) anxiety; (**b**) depression; (**c**) well-being; (**d**) self-control; (**e**) general health; (**f**) vitality. Classes of percentile were defined for subscales, according to gender and age, as follows: <25th pc: ‘severe distress’; between 25th and 50th percentile: ‘moderate distress’; between 50th and 75th percentile: ‘no distress’; >75th percentile: ‘positive well-being’. Red columns indicate PCD patients and grey columns indicate healthy controls. No significant difference between the number of PCD patients and controls with subscale scores <25th percentile was found (a–f). Fewer PCD subjects than controls reported (a) anxiety, (b) depression, (c) difficulties relating well-being or (f) vitality feelings, whereas more difficulties were with (d) self-control and (e) general health were found in a higher proportion of PCD cases than in controls.

As shown in Table 3, results of the PGWBI questionnaire showed that 13/17 cases (76%) of Group B participants did not present total scores indicating distress. In 3/17 participants (18%), the total score indicated ‘moderate distress’ and only in 1/17 participant (6%) ‘severe distress’. In the control group, 12/17 participants (70%) did not present total scores indicating distress, while 3/17 (18%) and 2/17 (12%) of the remaining cases showed a total score of moderate and severe distress, respectively. Percentages of patients and controls with different categories of PGWBI (i.e., severe; moderate; no distress) were not significantly different (*p* > 0.05). Compared to controls, in PCD participants aged ≥15 years, no significant differences of raw scores of anxiety, depression, well-being, self-control, general health, vitality, and PGWBI total index were found (*p* > 0.05; Figure 2). significant difference was found between the number of PCD patients and controls with subscale scores <25th percentile (indicating severe distress) (Figure 3a–f). However, fewer PCD subjects than controls reported anxiety, depression, difficulties relating well-being or vitality feelings (Figure 3a–c,f), while more difficulties associated with self-control and general health were found in a higher proportion of PCD cases than controls (Figure 3d,e).

Compared to the rate in the same period of the current year (2020), the pulmonary exacerbation rate was higher in the period February–4 May 2019 in the whole PCD study population, (*p* = 0.03), and in PCD subjects aged <15 years (*p* = 0.02) or aged ≥15 years (*p* = 0.04), respectively (Table 4). The mean number of chest physiotherapy sessions per week performed by each PCD patient significantly increased during the quarantine period (i.e., from 8 March to 4 May 2020) compared to the same period in the previous year (12 versus 8 sessions/week per patient, respectively; *p* = 0.04).

## 7. Discussion

To our knowledge, this study is the first prospective evaluation on the levels of psychological burden in a PCD population compared to healthy subjects during the imposed lockdown period for COVID-19 in Italy. PCD is a chronic disorder with potential psychological effects on the intra-familiar relationships because of patients’ frequent need of medical consultations, their own perception of being sick, and the possible effects of denial and rejection of the disease by patients and/or parents. Our main finding is that, overall, during the COVID-19 quarantine, PCD patients did not show scores indicating distress, and only some of them reported more difficulties related to self-control and general health. Among PCD parents, only 20% showed high levels of stress. Finally, compared to healthy subjects, the current PCD population did not show significantly different psychological burden or parental stress level during the quarantine. Patients with PCD and their families, as well as controls, strictly observed the stringent preventive measures ordered by the Italian government. It is possible to suppose that the quarantine, avoiding the risk of exposure to SARS-CoV-2, gave PCD patients a great sense of security. Despite the fact that the access to the healthcare service was fairly limited, the telemedicine facility at our hospital warranted a continuous medical surveillance through remote contact.

Some data from the current study deserve a comment. The finding of less PCD airway infectious exacerbations during the COVID-19 quarantine period imposed in Italy, quite novel to the best of our knowledge, is not surprising, and might be likely explained by the strict adherence to the preventive measures for reducing viral transmission. The preventive measures consisted of (a) social distancing with avoidance behaviors; (b) the adoption of the smart working model that allowed workers to work at home with a flexible time schedule; (c) schools closure for limiting contacts with or among kids and their teachers; (d) education to frequent handwashing; (e) respiratory and environmental hygiene measures (especially, while coughing or sneezing) [25]. Since healthcare institutions are major foci of disease, and the nosocomial SARS-CoV-2 transmission is a concrete possibility, several hospitals, including ours, limited the access to healthcare services during the quarantine unless urgent or serious problems were claimed. In PCD, the detection of respiratory pathogens in the lower airways is associated with frequent exacerbations, and it fuels the vicious cycle of infection, inflammation, and lung damage that further increases bacterial growth [8]. Therefore, the absence of stress perception during the quarantine found in the vast majority of our study population might be related to fewer symptoms or signs of pulmonary exacerbation. As our patients during the COVID-19 quarantine period had a lot more free time, we speculate that the smart working model or school closure likely resulted in improved compliance to chest physiotherapy. Therefore, quarantine might explain, at least in part, the reduced number of airway infections [26].

In PCD, these infections are due to retention of airways mucus and growth of biofilms caused by abnormal ciliary structure and/or function. The diagnostic delay and delayed start of PCD treatment may eventually result in patients’ uncertainty or anxiety about their disease outcome. Patients’ emotional status may have a negative impact and patients and their families may be at risk for impaired psychosocial functioning. Research on well-being and psychological issues of PCD has focused on the emotional burden of the condition, including patients’ concern about current and future health [27]. Several studies have highlighted that PCD patients of any age are at risk of experiencing anxiety or depression, or reduced self-esteem, even though they may not present worse rates of psychological well-being than healthy peers [28,29,30].

The primary goal of public health measures for controlling a pandemic is to prevent person-to-person spread of the infection by separating people. Isolation is an unpleasant experience, and individuals report boredom or a sense of frustration due to reduced social contacts [31]. During disease outbreaks, anxiety can also rise following media reporting on new cases or deaths rates [32]. Moreover, being unable to receive regular medical care and prescriptions may be a matter of concern, especially for patients with chronic disorders [33,34]. In PCD patients and their families, the uncertainty about the future, combined with the fear that any medical problem could not be addressed because hospital visits were blocked might increase patients’ or parents’ levels of anxiety. However, social distancing likely halted the transmission of all respiratory pathogens also including SARS-CoV-2, with a beneficial effect on PCD patients and family well-being.

Stress levels of parents of PCD patients aged<15 years were evaluated rather than the patients themselves. Indeed, the remote assessment of the psychological state in younger participants could be unreliable because of the difficulties of self-monitoring and self-awareness at that age. Actually, although a great emotional and stress load was reported in PCD [30], during COVID-19 quarantine, stress level was not increased compared to the healthy peers. Nevertheless, in the current PCD mothers’ group, the PSI-SF total score and the scores of other subscales was higher than in the control group, although the difference was not statistically significant. The data suggest that, in Italy, the emergency condition associated with COVID-19 increased the stress levels in the general population [35], reducing the gap with subjects with an underlying chronic condition, such as PCD. Actually, greater care, greater hygiene, and staying at home might represent a protective factor for people with chronic respiratory diseases, paradoxically affecting the levels of psychological well-being in their parents. Comparing the scores obtained in each subscale, it was found that the percentage of subjects with clinically relevant scores of the “Difficult Child” subscale, was greater in the group of PCD mothers than in the control group. Moreover, 30% of PCD mothers reported *t*-scores above 85th percentile in the subscale relating to DC. The DC subscale represents a parent’s perception of his/her child, seen as a ‘difficult child’ for any problematic behavior. High scores on this scale indicate the presence of characteristics that may limit parents in their role. This finding suggests that some of PCD mothers felt themselves more stressed during the quarantine period in the daily management of their kids compared to the mothers of healthy kids.

The evaluation of the psychological impact of the COVID-19 quarantine did not reveal clinically significant differences in PCD patients older than 15 years compared to the control group. Nevertheless, comparing the scores obtained for each subscale, the percentage of subjects with clinically relevant scores associated to ‘anxiety’, ‘depression’, ‘positivity’, ‘well-being’, and ‘vitality’, was greater in the control group than in PCD patients. Conversely, more PCD subjects showed clinically significant scores in the subscales related to ‘self-control’ and ‘general health’. These data indicate that anxiety and depression levels in the healthy controls were increased because of the concerns of the outbreak, with a consequent reduction in the perception of well-being and vitality, as recently reported [36,37,38,39,40]. In PCD patients, on the other hand, self-control was decreased, perhaps because of the fear of getting infected. The general sense of health might be perceived as more precarious, but stress levels, mood, and perception of anxiety were stable.

The study has strengths and limitations. First, novel information of the psychological effects of the quarantine in PCD patients was provided, that was lacking in the literature. An additional strength is the availability of detailed medical records from a well-defined cohort of PCD patients. These records allowed to compare the data collected during the COVID-19 lockdown period to the same period of the previous year. Moreover, we designed a prospective, observational study with a control group that adhered strictly to the isolation preventive measures as well as PCD patients. Yet, the study had some limitations. First, due to the unexpected nature of the COVID-19 outbreak, the psychological burden in the previous year could not be evaluated and, thus, ignored the level of emotional stress in the pre-COVID-19 period. Second, the study population from a single-center in Southern Italy was small. Actually, designing a study of a large and homogeneous population of patients with a rare condition like PCD is not easy. However, these findings should hopefully be replicated by the results from larger PCD population in countries with even different sociocultural background.

## 8. Conclusions

In conclusion, this study provides the novel information that patients with PCD, a chronic condition with pre-existing potential characteristics of personal and familiar increased stress, during COVID-19 quarantine neither developed psychological distress nor experienced a psychological burden significantly different than controls. Similar multicenter, longitudinal studies in patients with underlying chronic respiratory disorders would increase the understanding of COVID-19 outbreak, which is the most dramatic experience lived through by humans during the last century.

## Figures and Tables

**Table 1:** Demographic and clinical characteristics of patients with primary ciliary dyskinesia.

Variables	Total (n = 27)	Group A (n = 10)	Group B (n = 17)
Male, no. (%)	15 (56)	6 (60)	9 (53)
Age, years * (SD)	22.6 (12.3)	12.8 (2.4)	28.4 (12.1)
Age at diagnosis, years * (SD)	7.7 (10.3)	2.6 (3.9)	10.8 (11.7)
Patients with laterality defect, no. (%)	22 (81)	7 (70)	15 (88)
Patients with bi-allelic mutation, no. (%)	23 (85)	8 (80)	15 (88)
Patients with abnormal TEM, no. (%)	17 (63)	6 (60)	11 (65)
FVC, % predicted * (SD)	94.7 (17.4)	96.1 (14.8)	94 (19)
FEV_1_, % predicted * (SD)	84.7 (21.6)	88.6 (21)	82.9 (22.3)
Patients with bronchiectasis, no. (%)	15 (56)	4 (40)	11 (65)
Monolateral, no. (%)	7 (26)	1 (10)	6 (35)
Bilateral, no. (%)	8 (30)	3 (30)	5 (29)
Patients with PA colonization, no. (%)	8 (30)	2 (20)	6 (35)

Abbreviations: n., number; SD, standard deviation; TEM, transmission electron microscopy; FVC, forced vital capacity; FEV_1_, forced expiratory volume in 1 s; PA, *Pseudomonas aeruginosa*. * Expressed as mean.

**Table 2:** Results of PSI-SF questionnaire administered to the mothers of Group A patients and controls.

Variables	Group A (n = 10)	Healthy Controls (n = 10)	*p*
Male	6 (60) †	7 (70) †	1.0
Age, years	12.8 (2.4) *	10.3 (3) *	0.56
**PSI-SF categories**			
High stress level (s > 85th percentile)	2 (20) †	0 (0) †	0.47
Symptomatic stress (50th > s < 85th percentile)	1 (10) †	1(10) †	1.00
Non-pathological stress (s < 50th percentile)	7 (70) †	9 (90) †	1.00
**PSI-SF scores**			
PD	23.8 (7.8) *	20.1 (6.0) *	0.12
PCD-I	20.3 (10.3) *	16.3 (4.4) *	0.27
DC	26.1 (9.6) *	19.8 (5.2) *	0.08
DEF	14.9 (3.7) *	13.0 (3.5) *	0.25
Total stress index	67.3 (23.0) *	53.9 (13.8) *	0.13

Abbreviations: s, score; PSI-SF, Parenting Stress Index- Short Form; PD, parental distress domain; P-CDI, parent-children dysfunctional interaction domain; DC, difficult child subscale; DEF, defensive responding. † Expressed as number of patients or controls and percentage in parenthesis. * Expressed as mean and Standard Deviation in parenthesis.

**Table 3:** Results of the PGWBI questionnaire administered to Group B patients and controls.

Variables	Group B (n = 17)	Healthy Controls (n = 17)	*p*
Male	9 (53) †	11 (65) †	0.73
Age, years	28.4 (12.1) *	34.5 (12.6) *	0.16
**PGWBI categories**			
Severe distress (s < 60)	1 (6) †	2 (12) †	1.00
Moderate distress (60 > s < 72)	3 (18) †	3 (18) †	1.00
NO distress (s > 72)	13 (76) †	12 (70) †	1.00
**PGWBI scores**			
Anxiety	17.5 (3.5) *	17.1 (3.5) *	0.77
Depression	12.8 (1.3) *	12.1 (1.4) *	0.11
Well-being	11.5 (3.0) *	10.8 (2.7) *	0.48
Self-control	11.8 (2.3) *	12 (2.2) *	0.82
Health	11.3 (1.5) *	11.8 (2.2) *	0.42
Vitality	14.3 (2.8) *	13.4 (2.6) *	0.32
PGWBI total score	79.2 (11.5) *	77.2 (12.2) *	0.62

Abbreviations: s, score; PSGWBI, psychological general well-being index. † Expressed as number of patients or controls and percentage in parenthesis. * Expressed as mean and standard deviation in parentheses.

**Table 4:** Pulmonary exacerbations in PCD patients during COVID-19 outbreak compared to the same period of 2019.

	February–May 2019	February–May 2020	*p*
Total patients (n = 27) Number of exacerbations (IQR)	1 (0–1)	0 (0–0)	0.03
Patients with at least one exacerbation, no. (%)	16 (59)	3 (11)	0.00
Patients aged <15 years (n = 10) Number of exacerbations (IQR)	1 (0–1)	0 (0–0)	0.02
Patients with at least one exacerbation, no. (%)	7 (70)	1 (10)	0.02
Patients aged ≥15 years (n = 17) Number of exacerbations (IQR)	1 (0–1)	0 (0–0)	0.04
Patients with at least one exacerbation, no. (%)	9 (53)	2 (12)	0.02

Abbreviations: n, number; IQR, interquartile range; PCD, primary ciliary dyskinesia.

## Data Availability

The datasets analyzed during the current study are available from the corresponding author on reasonable request.

## List of Abbreviations

PCD	Primary Ciliary Dyskinesia
SARS-CoV-2	severe acute respiratory syndrome due to coronavirus 2
COVID-19	Coronavirus Disease 2019
PSI-SF	Parenting Stress Index-Short Form
PD	Parental Distress
P-CDI	Parent-Child Dysfunctional Interaction
DC	Difficult Child
DEF	Defensive Responding
PGWBI	Psychological General Well-Being Index
TEM	transmission electron microscopy
FVC	forced vital capacity
FEV_1_	forced expiratory volume in 1 s
PA	*Pseudomonas aeruginosa*
